# Sugar regulation of *SUGAR TRANSPORTER PROTEIN 1* (*STP1*) expression in *Arabidopsis thaliana*

**DOI:** 10.1093/jxb/eru394

**Published:** 2014-10-03

**Authors:** Elizabeth Cordoba, Denise Lizeth Aceves-Zamudio, Alma Fabiola Hernández-Bernal, Maricela Ramos-Vega, Patricia León

**Affiliations:** Instituto de Biotecnología, Universidad Nacional Autónoma de México, Av. Universidad 2001, Col. Chamilpa, Cuernavaca, Morelos. México. C.P. 62210, Mexico

**Keywords:** *Arabidopsis*, STP1, sugar repression, sugar signalling, sugar transporter, transcriptional regulation.

## Abstract

Sugars regulate the expression of many genes at the transcriptional level. In *Arabidopsis thaliana*, sugars induce or repress the expression of >1800 genes, including the *STP1* (*SUGAR TRANSPORTER PROTEIN 1*) gene, which encodes an H^+^/monosaccharide cotransporter. *STP1* transcript levels decrease more rapidly after the addition of low concentrations of sugars than the levels of other repressed genes, such as *DIN6* (*DARK-INDUCED 6*). We found that this regulation is exerted at the transcriptional level and is initiated by phosphorylatable sugars. Interestingly, the sugar signal that modulates *STP1* expression is transmitted through a HEXOKINASE 1-independent signalling pathway. Finally, analysis of the *STP1* 5′ regulatory region allowed us to delimit a region of 309bp that contains the *cis* elements implicated in the glucose regulation of *STP1* expression. Putative *cis*-acting elements involved in this response were identified.

## Highlight text

Transcriptional repression of *STP1* is rapidly induced by phosphorylatable sugars through an HXK1-independent signalling pathway involving the participation of sugar-responsive *cis* elements localized in the promoter.

## Introduction

As autotrophic organisms, plants produce their carbon skeletons through the photosynthetic process in the form of sugars. These carbon skeletons are essential as structural components and energy sources for plant growth and development. Similarly to many other organisms, plants respond to the carbon fluctuations caused by changes in photosynthetic efficiency or metabolic status and adjust their growth and development accordingly (Baena-Gonzalez and Sheen, 2008; Nunes-Nesi *et al.*, 2010; Eveland and Jackson, 2012). Sugars act as signalling molecules, and plants have evolved mechanisms to efficiently perceive sugar availability and respond by modulating gene expression and protein activity in response to their nutrient status (Gibson, 2005; Rolland *et al.*, 2006; Smeekens *et al.*, 2010). The presence of sugars induces different developmental programmes, including growth, starch biosynthesis, and cell division. In contrast, sugar starvation upregulates photosynthetic activities and carbon remobilization, thus affecting central aspects of development (Koch, 1996; Borisjuk *et al.*, 2003). Thus, understanding the mechanisms involved in sugar perception form an important area of research.

Plants have the capacity to sense different sugars, including sucrose, hexoses, and trehalose, and elicit responses, some that are specific to the type of sugar (Chiou and Bush, 1998; Sheen *et al.*, 1999; Eastmond and Graham, 2003; Price *et al.*, 2004; Wind *et al.*, 2010). However, hexoses appear to be the most common signal detected by plants (Rolland *et al.*, 2006; Smeekens *et al.*, 2010). Diverse lines of evidence have demonstrated that sugar levels are detected by specific receptors and through independent signalling pathways (Rolland *et al.*, 2006; Hanson and Smeekens, 2009). One of those receptors is the HEXOKINASE 1 (HXK1) that, in addition to its enzymatic activity, acts a sugar sensor (Jang *et al.*, 1997; Moore *et al.*, 2003; Cho *et al.*, 2006). Experimental evidence has shown that upon sugar phosphorylation, HXK1 interacts with the VHA-B1 and RPT5B proteins to control the transcription of an important number of target genes (Cho *et al.*, 2006). Evidence exists for additional sugar receptors, including sugar transporters and the negative regulator of trimeric G-protein (RGS1), but their mechanisms of action and downstream components are still poorly understood (Chen and Jones, 2004; Rolland *et al.*, 2006).

Some of these sensors and downstream components of the sugar signalling pathways have initially been identified through genetic approaches with the isolation of sugar-response mutants. The characterization of these mutants has demonstrated the complexity of sugar signalling and the extensive cross-talk with other signalling pathways (León and Sheen, 2003; Gibson, 2005; Rolland *et al.*, 2006; Eveland and Jackson, 2012). Additional components that are required for proper sugar perception were identified from sugar-insensitive mutants, including the enzyme ABA2, the transcription factors ABI4 and ABI5, and the ethylene-insensitive EIN2 protein (Zhou *et al.*, 1998; Arenas-Huertero *et al.*, 2000; Cheng *et al.*, 2002). These factors were originally identified as components of the abscisic acid (ABA) or ethylene biosynthesis and signalling pathways, demonstrating the cross-talk between sugars and these hormones (Finkelstein and Gibson, 2002; León and Sheen, 2003; Yamagishi *et al.*, 2009). Cross-talk has also been reported between sugar signalling and other hormones, such as auxin and gibberellins (Moore *et al.*, 2003; Eveland and Jackson, 2012). Sugar signalling not only interacts with hormones but also with other nutrients, such as nitrogen (Coruzzi and Bush, 2001) and with the energy and stress signalling responses through the participation of the SnRK1 and TOR complexes (Baena-Gonzalez *et al.*, 2007; Baena-Gonzalez, 2010; Xiong *et al.*, 2013).

Based on the genes regulated by different sugars and sugar analogues, several pathways for sugar signalling are recognized and can be grouped into those that depend on HXK1 for signal initiation and those that are independent of this sensor (Rolland *et al.*, 2006). The last group includes several pathways, such as the SnRK1-mediated pathway (Baena-Gonzalez *et al.*, 2007; Jossier *et al.*, 2009), the RGS1 pathway and most likely other undiscovered pathways (Chen and Jones, 2004; Chen, 2008; Sheen, 2010). Due to the complexity of sugar signalling, alternative strategies are required to further understand the molecular basis and additional components of the different sugar signalling pathways.

The regulation of gene expression is one of the most prominent mechanisms by which sugars modulate a variety of responses in plants. Independently of the signalling pathway, sugars positively or negatively affect the transcription of nearly 2000 different genes (Wang *et al.*, 2003; Price *et al.*, 2004; Li *et al.*, 2006; Müller *et al.*, 2007). In spite of the number of genes regulated by sugars, only a few transcriptional factors are known to be involved in this regulation. In fact, the participation of several bZIP and MYB transcription factors was recognized through the use of novel screenings (Rolland *et al.*, 2006; Hanson and Smeekens, 2009; Sheen, 2010). The analysis of target genes has also proven to be a useful approach for identifying the *cis*-acting elements and *trans*-acting factors that are involved in sugar regulation. For example, using the promoter region of the α-amylase (*α-Amy3*) gene from rice allowed the identification of different MYB transcription factors that participate in the sugar regulation mediated by SnRK1A (Lu *et al.*, 2007).

To further elucidate the mechanisms underlying sugar signalling in plants, we characterized the regulation by sugars of the *Arabidopsis thaliana STP1* gene (AT1G11260). *STP1* encodes a high-affinity sugar transporter that acts as an H^+^/monosaccharide cotransporter, capable of transporting a wide range of hexoses (Boorer *et al.*, 1994; Büttner and Sauer, 2000). STP1 belongs to a family of 14 members that are highly conserved among plants and mediate hexose transport in cells of different tissues (Stadler *et al.*, 2003; Slewinski, 2011). Several of these transporters are expressed in a tissue-specific manner, or at specific developmental stages (Büttner, 2010). *STP1* is the member of the *STP* family with the highest expression level (Johnson *et al.*, 2006; Johnson and Thomas, 2007). This high expression is detected in photosynthetic tissues, such as leaves and stems, while roots, siliques, and flowers show lower expression levels (Sherson *et al.*, 2003). The expression of this transporter was also detected in guard cells and its accumulation responds to diurnal fluctuation that correlates with the accumulation of sucrose in this cell type. This has led to speculation that this H^+^/sugar cotransporter might be important for osmoregulation during the day and night periods (Stadler *et al.*, 2003). The expression of several members of the *STP* family, including *STP1*, *STP4*, *STP13*, and *STP14,* is strongly repressed by sugars, and *STP1* is one of the most repressed genes, as indicated by wide-genome analyses (Price *et al.*, 2004; Büttner, 2010;). However, neither the pathway implicated in this regulation nor the factors involved are known. In this work, we analysed the mechanism that regulates the expression of the *STP1* gene in response to sugar levels. This analysis demonstrated that the *STP1* transcript is strongly downregulated within minutes after sugars increase. Interestingly, the regulation of this gene by sugars depends on phosphorylated hexoses but is independent of HXK1. We demonstrated that the regulation of this gene occurs at the transcriptional level and that the *cis*-acting elements responsible for this regulation are within a 309bp fragment of the promoter.

## Material and methods

### Plant material and growth conditions

For sterile growing conditions, *A. thaliana* seeds were sterilized following standard protocols (http://www.arabidopsis.org/). To break dormancy, the seeds were incubated for 3 days at 4°C in darkness. Adult plants were grown in Metro-Mix 200 (Grace Sierra, Milpitas, CA, USA). Plants and seedlings were grown under a 16h light/8h dark cycle in 120 µM m^2^ s^–1^ light conditions at 22°C. Wild-type Col-*0* and the *gin2*, *abi4-1*, *abi5, kin11, rgs1*, and *rgs1-2* mutants were obtained from the Arabidopsis Biological Resource Center (http://www.arabidopsis.org/). The *KIN10* knockout mutant and overexpressing lines were kindly donated by Dr Phillip Rolland (Institute of Botany and Microbiology, Heverlee-Leuven, Belgium). For sugar gene expression analysis, 50 seeds per treatment were grown in liquid 0.1X GM medium containing Murashige and Skoog basal salts (Caisson Laboratories Inc., UT, USA), supplemented with B5 vitamins (Sigma In., MO, USA), 0.05% MES, and 0.5% sucrose, and maintained with agitation at 350rpm. After 10 d, this medium was replaced with carbon (-C) starvation medium (0.1X GM without sucrose) for 2 d. Finally, the treatments were applied in 0.1X GM with or without sugar as indicated in each case using D-glucose monohydrate (Research Organics Cleveland, USA) or mannitol (Sigma-Aldrich, MO, USA) as carbon sources, as indicated.

Transgenic lines were generated through *Agrobacterium tumefaciens*-mediated transformation by floral dipping (Clough and Bent, 1998) into the Col-*0* ecotype. Transgenic lines were selected in 1X GM media with 0.8% Phytagar and supplemented with 50 µg ml^–1^ kanamycin. At least three independent lines were selected for each construct.

### Plasmid constructions

To generate transcriptional fusions of the *STP1* upstream region with the GUS reporter gene (Jefferson *et al.*, 1987), the 2.4kb fragment of the intergenic region of *STP1* (between the loci AT1G11250 and AT1G11260) was amplified by PCR from DNA using the oligonucleotides STP1-3, 5′-AAG CTT CTC TGA CTG ACG TTA AAT TC-3′, and STP1-5R, 5′-GGA TCC TAA ACA AGA CCC GTA AA-3′. The 1.3kb, 1kb, and 0.5kb deletions were generated from the original 2.4kb fragment through PCR using the following specific forward oligonucleotides: STP1-1327F, 5′-CCA ATG CGG CCG CCC ATG AAA C-3′; STP1-1HF, 5′-GTT GAA GCT TTA GAG CAC TAT G-3′; and STP1-527HF, 5′-GCA AGC TTG TTT CAC ATT TTA AC-3′; and the common reverse STP1-5R oligonucleotide. All the fragments were cloned into the TOPO-TA vector (Invitrogen, Carlsbad, CA, USA) and confirmed by sequencing. Each fragment was subcloned into the pBI101 vector binary vector (Clontech Laboratories, Inc. CA, USA) in the *Hind*III and *Bam*HI restriction site.

### Expression analysis

Total RNA was isolated from frozen tissue using TRIzol (Invitrogen, Carlsbad, CA, USA) according to the protocol provided by the manufacturer. For northern blot analyses, 1–20 µg of total RNA was fractionated in 1.5% agarose denaturing gels with 2% formaldehyde (Mallinckrodt Baker, MEX) and transferred onto a Hybond-N^+^ nylon membrane (GE, Bucks, UK). Hybridizations and washes were performed in stringent conditions. Probes were ^32^P-radiolabelled using the Megaprime DNA labelling system, according to the manufacturer’s protocol (GE, Bucks, UK). All probes were obtained by PCR amplification from DNA or cDNA as indicated. The *STP1* (At1g11260) probe corresponds to a cDNA fragment of 499bp that was obtained using the oligonucleotides STP1-1, 5′-TGC TAT AGT GGT TGT AAC GTT CAT T-3′, and STP1-2, 5′-GGC TAA TAC ACT TTT TCC TTT ACG ACA-3′. The GUS probe (860bp) was obtained using the oligonucleotides GUS3F, 5′-CGA AAA CTG TGG AAT TGA TCA G-3′, and GUS4R, 5′-ACC ATC AGC ACG TTA TCG AAT C-3′. For *DIN6* (At3g47340), a 382bp fragment was obtained using the oligonucleotides DIN6F, 5′-GCC TGA AAG ATC ACG CTG CTC-3′, and DIN6R, 5′-GCC TTT GCA GTC GAA CAA GCC-3′. For *β-AMY* (At4g15210), a 608bp cDNA fragment was obtained using the oligonucleotides β-Amy-1, 5′-CGG AGA AGG GGA AGT TTT TC-3′, and β-Amy-2, 5′-AAT CTC ATG CCC GTA CTT CG-3′. For *SBE2.2* (At5g03650), a 335bp cDNA fragment was obtained using the oligonucleotides SBE2.2A, 5′-GAG TGT CTC TTA CTC CAC GC-3′, and SBE2.2B, 5′-GGG AAC TAT TCT TGG TTT CAC-3′. For *APL3* (At4g39210), a 345bp cDNA fragment was obtained using the oligonucleotides Apl3-1, 5′-TTC TTG GGA GAA TGC AGC ATC-3′, and Apl3-2, 5′-TGT TCA TAT CAC AGT ACC GTC-3′. Densitometric analysis was performed using the ImageJ 1.43u program from the National Institutes of Health, USA, http://rsb.info.nih.gov/ij. To evaluate confidence of the data we used ANOVA statistical analysis (http://www.r-project.org/).

### GUS histochemical analysis

Twelve-day-old seedlings exposed to different sugar sources for 12h were stained for 2h using the GUS histochemical assay as reported (Jefferson *et al.*, 1987). Plant were clarified using a modified protocol from Malamy and Benfey (1997). Pigments were removed with 70% ethanol and plants were rehydrated by incubations in 50% and 30% ethanol for 15min each, and then transferred to a solution of 0.24 N HCl in 20% methanol and incubated at 62ºC for 1h. This solution was replaced by 7% NaCl in 60% methanol and incubated for 25min at room temperature. Then plants were dehydrated with 40%, 20%, and 10% ethanol for 15min each to finally be mounted in a solution with 50% glycerol and 2% DMSO. Samples were visualized using a stereoscopic (Nikon SMZ1500) and a light microscope (Nikon Eclipse E600).

### *In silico* analysis

The 309bp sequence from the *STP1* promoter was analysed using the PLACE (Plant *Cis*-acting Regulatory DNA Elements) database (Prestridge, 1991; Higo *et al.*, 1999), and released data were analysed to identify the reported motifs involved in sugar regulation. Additional *cis* elements, which were not included as sugar-responsive elements in this database, were identified by manual comparison with the promoter sequence for *DIN6* (At3g47340), a gene that is downregulated in the presence of sugar (Li *et al.*, 2006; Baena-Gonzalez *et al.*, 2007).

## Results

### *STP1* expression is rapidly regulated by glucose supply

**Fig. 1:**
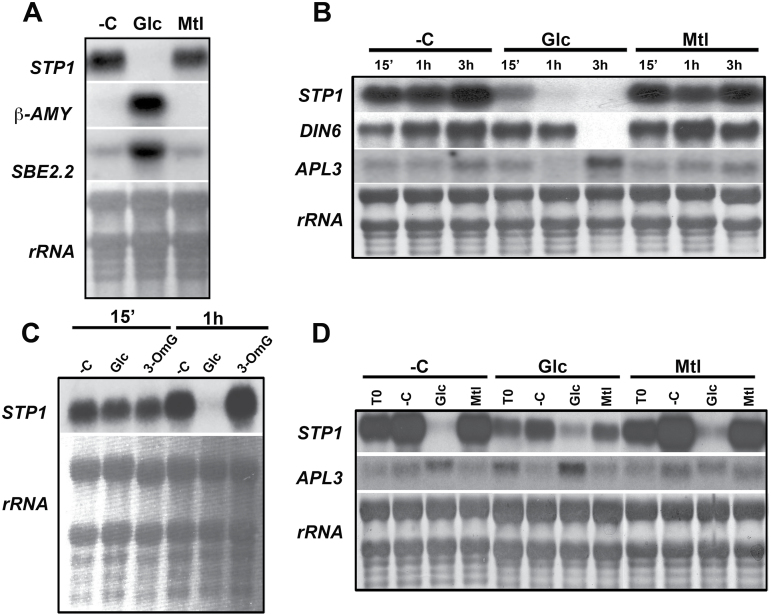
*STP1* expression is repressed by glucose. (A) Northern blot analysis of the total RNA from 12-day-old Col-*0* plants that were transferred to media without a carbon source (–C) or with 150mM glucose (Glc) or 150mM manitol (Mtl) for 6h. (B) Time course of the *STP1* expression of plants exposed to media without carbon (–C) or with 150mM Glc or 150mM Mtl for 15min, 1h, and 3h. (C) *STP1* expression from plants that were treated with 5mM Glc or 3-*O*-methylglucose (3-OmG) for 15min or 1h. (D) Transcript expression profile from 12-day-old plants grown for 2 days in 0.1X GM without a carbon source (–C) or with 150mM Glc or Mtl and then transferred for 6h to –C medium or medium supplemented with 150mM Glc or Mtl. Each lane in the different blots contains 10 µg of total RNA. The blots were hybridized with radioactive probes for *STP1*, *β-AMY* (beta-amylase)*, SBE2.2* (starch-branching enzyme 2.2), and *APL3* (ADP-glucose pyrophosphorylase large subunit), as indicated. The rRNA from the methylene blue-stained membranes is shown as a loading control. The membranes shown are representative of at least two biologically independent experiments.

Analysis of the available microarray data indicated that *STP1* (AT1G11260) was one of the most prominent downregulated genes in response to sugars in *A. thaliana* (Price *et al.*, 2004). Thus, we wanted to characterize the regulatory mechanism involved in the *STP1* gene response to sugars. To corroborate the effect of sugars on *STP1* expression, we analysed the accumulation of its transcript in the presence of glucose (Glc) by northern blotting. Sugar treatments were performed after carbon starvation in liquid media (see Material and Methods). As shown in Fig. 1A and in agreement with published microarray data, the *STP1* transcript level was dramatically reduced in the presence of 150mM Glc, relative to the levels in the untreated samples (–C) or in the isosmotic control with mannitol (Mtl). Under these conditions, the expression was induced (Fig. 1A) for the known Glc upregulated genes, such as *β-AMY* (*Beta-amylase*) and *SBE2.2* (*Starch Branching Enzyme 2.2*), that were used as controls (Rook *et al.*, 2001). These results confirm that the *STP1* transcript is downregulated by the presence of Glc (Price *et al.*, 2004).

For more detailed analysis of the regulation of the *STP1* gene in response to sugar, the level of its transcript was followed at different times after the addition of Glc. As shown in Fig. 1B, the *STP1* transcript level decreased 15min after the addition of Glc. This repression was not observed in the absence of external sugar (–C) or with the addition of Mtl. The *STP1* transcript was basically undetectable 1h after the treatment. The rapid response of *STP1* contrasts with the slower response for other characterized sugar-repressed genes, such as *DIN6*/*ASN1*, which encodes the asparagine synthetase 1 enzyme (Lam *et al.*, 1998; Price *et al.*, 2004). The reduction of the *DIN6* transcript was evident only 3h after the addition of Glc (Fig. 1B). A similar situation was observed for the Glc-upregulated *APL3* gene (encodes the large subunit of ADP-glucose pyrophosphorylase), whose transcript accumulation in response to Glc was detectable only 3h after the addition of sugar (Fig. 1B). These findings demonstrate that the expression of *STP1* is rapidly modulated by the changes in sugar levels.

To establish the sensitivity of *STP1* to sugars, the expression of this gene was analysed in the presence of different Glc concentrations (data not shown). As shown in Fig. 1C, the presence of 5mM Glc was sufficient to dramatically decrease *STP1* transcript levels, albeit after a longer time (1h) than the 15min that was found with 150mM Glc (Fig. 1B). This reduction was not observed with 3-*O*-methylglucose (3-OmG), a poorly metabolized Glc analogue (Fig. 1C). These data allowed us to conclusively demonstrate that the accumulation of the *STP1* transcript is rapidly downregulated by the presence of low Glc levels and that this regulation is not related to an osmotic response.

### *STP1* gene expression is dynamically regulated by sugar fluctuation

Experimental evidence has demonstrated that regulation by sugars is complex. For example, sugar regulation of the *α-Amy3* gene from rice involves both transcriptional repression and activation in response to the presence or absence of a carbon source. These positive and negative regulations involve the action of different *trans*-acting factors on the same *cis*-acting regulatory element (Lu *et al.*, 1998; Lu *et al.*, 2002). To further understand how *STP1* responds to changes in the carbon supply, the levels of its transcript were analysed in response to fluctuations in sugar availability. For this purpose, 10-day-old plants grown either in sugar starvation (–C) or in the presence of 150mM Glc or Mtl for 2 days were transferred to media without a carbon source (–C) or with 150mM Glc or 150mM Mtl for 6h, and the levels of the *STP1* transcript were analysed. In agreement with our previous results, the initial level of *STP1* transcript (T0) was lower in the plants grown in the presence of Glc than in those grown in its absence. Independently of the initial *STP1* transcript level, the addition of Glc resulted in a drastic reduction of the *STP1* transcript in the plant (Fig. 1D). In contrast, when the plants were transferred to media without sugar (–C), the *STP1* transcript accumulated (Fig. 1D). In addition, in accordance with published results, the expression of the *APL3* gene increased in the presence of Glc, albeit at different levels depending on the initial media in which the seedlings were grown prior to the treatment (Fig. 1D). Together, these data demonstrated that Glc regulation of *STP1* mRNA is rapid and dynamic. This result also indicates that multiple elements may be involved in the Glc regulation of *STP1*, a regulation that is similar to that of the rice *α-Amy3* gene.

### Sugar regulation of *STP1* responds to the level of phosphorylated hexose

**Fig. 2.**
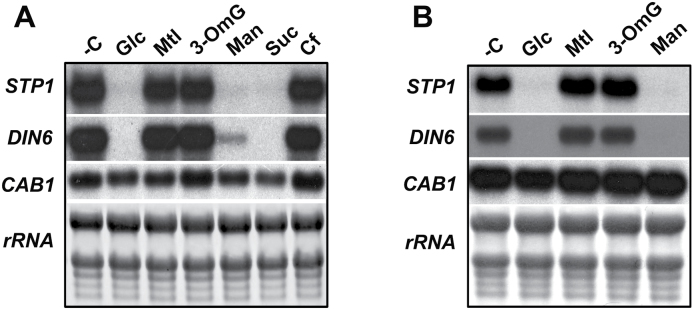
Regulation of *STP1* expression by sugars. The expression of *STP1*, *DIN6*, and *CAB1* was analysed by a northern blot from the RNA extracted from 12-day-old plants grown for 2 days in sugar-depleted (–C) media and then transferred to 150mM (A) or 5mM (B) Glc, Mtl, 3-OmG (3-*O*-methylglucose), Man (mannose), or Suc (sucrose) for 6h. Each lane was loaded with 10 µg of total RNA. The rRNA from the methylene blue-stained membrane is shown as a loading control. The blots shown are representative of three biologically independent experiments.

Due to the rapid and sensitive response observed in the accumulation of the *STP1* transcript in response to fluctuations in the Glc level, we decided to further investigate the mechanism involved in this regulation. Plants have the capacity to sense different sugars and transmit their signals through different pathways that involve specific components and mechanisms (Xiao *et al.*, 2000; Rolland *et al.*, 2006). The use of Glc analogues has been useful for characterizing the requirements for the regulation of specific genes by sugars (Jang and Sheen, 1994). Thus, we analysed the effect of the Glc analogues mannose (Man) and 3-OmG on *STP1* expression. In the presence of 150mM Man, low levels of the *STP1* transcript were observed, similar to those found with 150mM Glc (Fig. 2A). Man is a Glc analogue that is transported into cells and phosphorylated by hexokinase (HXK) but is very slowly metabolized (Jang and Sheen, 1994; Xiao *et al.*, 2000). In contrast, in the presence of 3-OmG, no repression of the *STP1* transcript was observed, and the expression level remained comparable to the one found in the carbon deprivation (–C) condition (Fig. 2A). 3-OmG is transported into the cell (Jang and Sheen, 1997; Lalonde *et al.*, 1999; Smeekens, 2000) but is not phosphorylated because it is a very poor substrate for HXK (Cortes *et al*., 2003). Finally, similarly to Glc, the addition of sucrose (Suc) resulted in very low *STP1* levels (Fig. 2A). The response observed for *STP1* to these different sugar analogues was the same with lower levels (5mM) of these sugars (Fig. 2B). Similar responses to these sugar analogues were found for the *DIN6* gene, which is also induced by sugar starvation (Baena-Gonzalez *et al.*, 2007). Under the conditions used in this analysis, only a slight reduction in the expression level of the photosynthetic *CAB1* gene was detected (Fig. 2), suggesting that the response of this gene requires a longer treatment time or higher sugar concentrations. Together, these results support the hypothesis that the signal that initiates the regulation of the *STP1* transcript requires a phosphorylatable hexose, such as Glc or Man.

### An independent HXK1 pathway drives the sugar regulation of *STP1*

**Fig. 3.**
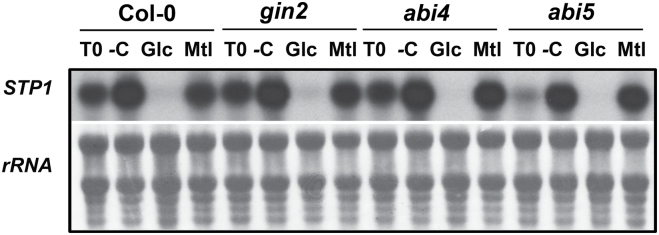
*STP1* regulation by Glc is mediated by an HXK1-independent signalling pathway. *STP1* expression was analysed in wild-type plants and in the sugar signalling mutants *gin2*, *abi4*, and *abi5*. Samples were obtained from 12-day-old plants grown for 2 days in sugar starvation (–C) conditions and then transferred to –C, 150mM Glc, or Mtl media for 6h. Ten micrograms of total RNA was used for northern analysis and was hybridized with the *STP1* probe. An initial control, which was measured before the treatments (T0), is included. The rRNA from the methylene blue-stained membranes is shown as a loading control. The membrane shown is representative of two biologically independent experiments.

Previous work has demonstrated that HXK1 functions as a primary Glc sensor that initiates a specific sugar signalling pathway; this pathway then induces or represses the expression of many genes in response to phosphorylated sugars (Moore *et al.*, 2003; Price *et al.*, 2004; Li *et al.*, 2006). Additional components of this signalling pathway include the ABI4 and ABI5 transcription factors (Arenas-Huertero *et al.*, 2000). To determine whether any of these components are required for *STP1* sugar regulation, we evaluated the *STP1* transcript level in response to Glc in the HXK1 (*gin2*), *abi4-1*, and *abi5* mutants. As shown in Fig. 3, the level of the *STP1* transcript in all three mutants decreased upon Glc addition, similarly to wild-type plants. No decrease in the transcript level was observed in the absence of sugar or in the corresponding Mtl isosmotic control (Fig. 3). In this analysis, we observed that the *STP1* transcript level in the *abi5* mutant prior to sugar treatment (T0) was lower than that of the wild type and the other mutants (Fig. 3). This result suggests that ABI5, independently of its role in sugar regulation, is required to maintain normal levels of the *STP1* transcript. However, these results demonstrated that neither HXK1 nor the transcription factors ABI4 or ABI5 participate in the sugar regulation of the *STP1* transcript.

**Fig. 4.**
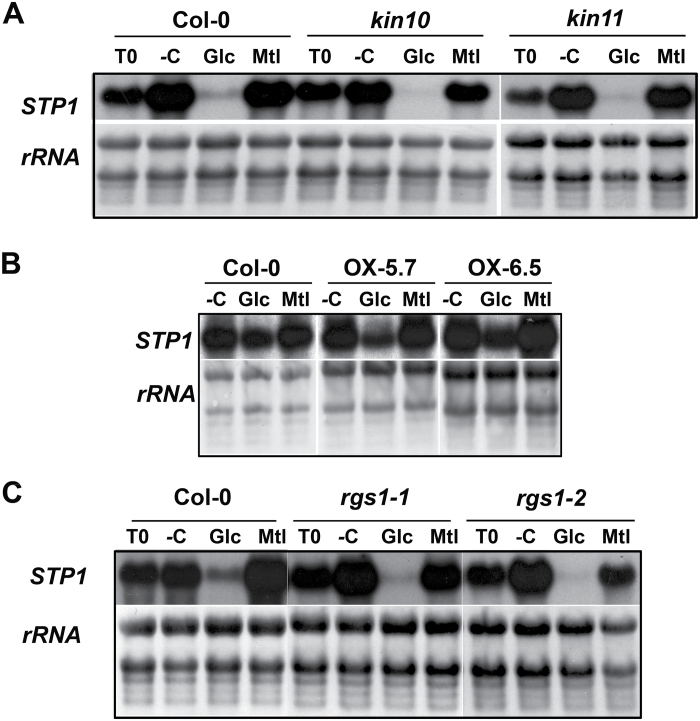
*STP1* sugar regulation in SnRK1 and RGS1 mutants. Total RNA was obtained from 12-day-old *kin10* and *kin11* mutants (A), from two independent overexpressing KIN10 (OX5.7 and OX6.5) lines (B), and from *rgs1-1* and *rgs1-2* (C). After carbon starvation for 2 days, the plants were transferred to media depleted of sugar (–C) or with 150mM Glc or Mtl for 6h (A and C) or with 5mM Glc or Mtl for 30 min (B). Ten micrograms of total RNA was used from each sample for northern analysis and was hybridized with the *STP1* probe. T0 represents the level of *STP1* prior to the sugar treatment. The rRNA from the methylene blue-stained membranes is shown as a loading control. Membranes shown are representative of two biologically independent experiments.

In addition to HXK1, other factors have been shown to play important roles in plant sugar responses, including SnRK1 kinase and a heterotrimeric G protein (Baena-Gonzalez and Sheen, 2008; Urano *et al.*, 2013). To analyse the possible role of these factors in the regulation of *STP1* by sugars, we measured the expression of *STP1* in the corresponding mutants. In the case of SnRK1 kinase, we evaluated knock-out mutants for the two catalytic subunits, *kin10* and *kin11.* Although these catalytic subunits are known to be partially redundant, analysis of a double mutant was not possible due to its lethality (Baena-Gonzalez and Sheen, 2008). In the case of the G protein, we evaluated two independent null mutant alleles of the RGS1 factor (*rgs1* and *rgs1-2*), a protein that modulates G-protein signalling and that has been reported to be an important component for HXK-independent sugar signalling responses (Chen *et al.*, 2003). As shown in Fig. 4A, no difference in the regulation of *STP1* by Glc was found in the *kin10* or *kin11* mutants compared to that in the wild-type Col-0 plants. However, due to the partial redundancy of these subunits, the participation of SnRK1 in the regulation of *STP1* in response to Glc cannot be totally excluded. To further address the function of the SnRK1 complex in the Glc regulation of the *STP1*, two independent lines that overexpress the KIN10 catalytic subunit (*KIN10*-OX) were analysed (Baena-Gonzalez and Sheen, 2008). We hypothesized that if the SnRK1 kinase has any role in the sugar repression of the *STP1* gene, this response should be exacerbated in the KIN10 gain-of-function lines. Because the level of the *STP1* transcript in the wild-type plants remain unaltered for the first 15min after the addition of 5mM Glc (Fig. 1C), we analysed the *STP1* level in two overexpressing lines, *KIN10*-OX5.7 and *KIN10*-OX6.5, under these conditions. However, we did not detect any difference in the *STP1* expression level between the overexpressing lines and the wild-type plants 15min after Glc addition (data not shown). We also analysed *STP1* levels after exposure to Glc for a longer time (30min). In this case, we detected a slight increase in the repression level in the overexpressing seedlings (Fig. 4B). Densitometric analysis of the *STP1* signal from independent biological experiments showed that repression of *STP1* expression in the *KIN10*-OX lines was 31%, compared to 22% in the wild-type plants. Finally, no difference was detected in the *STP1* sugar regulation in the two mutant alleles of the *RGS1* gene in comparison to that in the wild-type plants (Fig. 4C). These results suggest that none of the factors analysed here play a major role in the regulation of *STP1* by sugars.

### The regulation of *STP1* by sugars depends on the DNA elements located in the upstream region of this gene

**Fig. 5.**
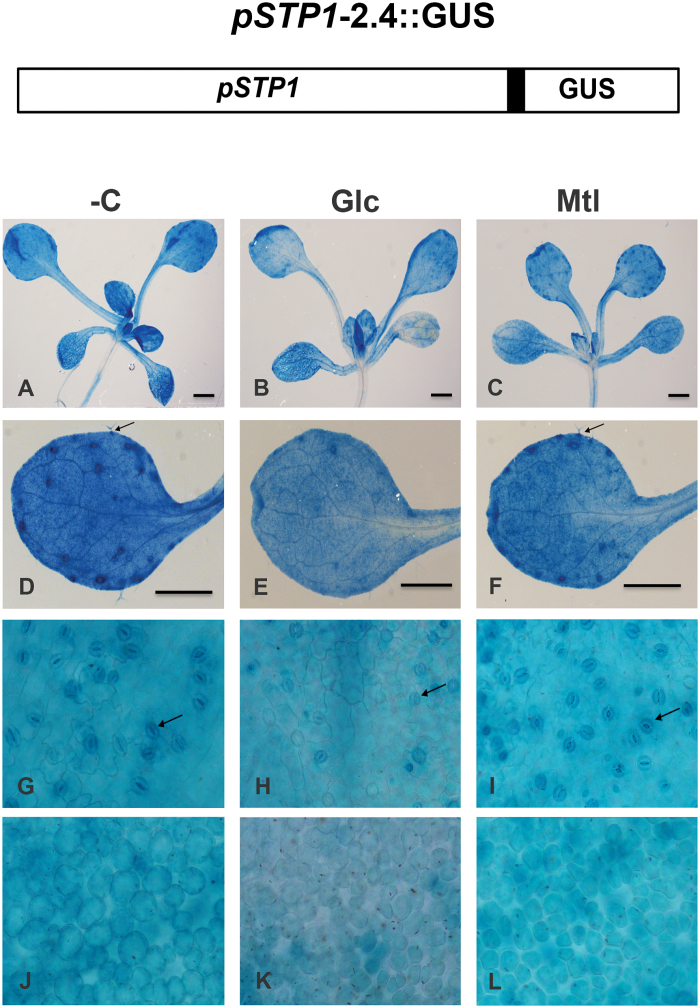
Expression pattern of the *STP1* gene in seedlings in the presence or absence of sugars. At the top is a diagram of the transcriptional fusion including 2.4kb of the 5’ upstream regulatory region of the *STP1* gene (p*STP1*-2.4kb) fused to the GUS reporter gene used to generate the transgenic lines. The corresponding 5’ UTR region is shown as a black box. The panels below show GUS staining, including the *pSTP1*:GUS expression pattern from a representative 12-day-old transgenic line exposed for 12h to media in the absence (–C) or presence of 150mM glucose (Glc) or mannitol (Mtl). Promoter expression pattern in the whole plant (A, B, and C) and in leaves from –C plants (D), with Glc plants (E), or with Mtl plants (F). Arrows mark the trichomes; the base of the mature trichomes strongly stained for GUS activity. (G–L) Epidermal surface of rosette leaves showing stomata (marked by arrows) from plants grown –C (G), with Glc (H), or with Mtl (I); and GUS activity in mesophyll tissue from plants grown –C (J), with Glc (K) and with Mtl (L). Bars correspond to 1mm.

Transcription plays a major role in the sugar regulation of many genes (Price *et al.*, 2004; Bläsing *et al.*, 2005). In various cases, this regulation depends on the presence of one or more *cis*-acting elements in the promoter region of the sugar-regulated genes (Chen *et al.*, 2006; Li *et al.*, 2006). However, post-transcriptional regulatory mechanisms are also involved in the regulation by sugars (Rolland *et al.*, 2006; Hummel *et al.*, 2009). To characterize the molecular mechanism of the regulation by Glc of the *STP1* gene, a 2.4kb fragment upstream from the ATG, which includes the upstream regulatory region and the 5′ UTR, was fused to the β-glucuronidase (GUS) reporter and introduced into *A. thaliana* plants (Fig. 5). Three independent transgenic lines (L1-2, L3-6, and L4-5) were selected, and the presence of the transgene was confirmed by PCR (data not shown). Homozygous plants from each line were selected.

GUS expression patterns of three independent transgenic lines were analysed in 12-day-old seedlings. GUS staining was prominently detected in leaves but it was also present in the vascular system of stems and roots with lower but clearly detectable levels (Fig. 5A). As previously published (Stadler *et al.*, 2003), the site of higher expression at this developmental stage was in leaves (Fig. 5A). A detailed analysis of this organ revealed that *STP1* expression was particular strong in trichomes, including the base of the stalk and the cells around them (Fig. 5D), and in stomata (Fig. 5G). The expression in the guard cells was not homogenous with a more intense GUS activity at the membrane near the stomatal pore (Fig. 5G). GUS staining was also present in the mesophyll cells of the leaves at lower levels (Fig. 5J). In contrast to published data (Stadler *et al.*, 2003), we could not detect differences in the GUS expression between the adaxial or abaxial surfaces of the leaves. However, such a difference might exist but be masked by the diffusion of the GUS marker.

**Fig. 6.**
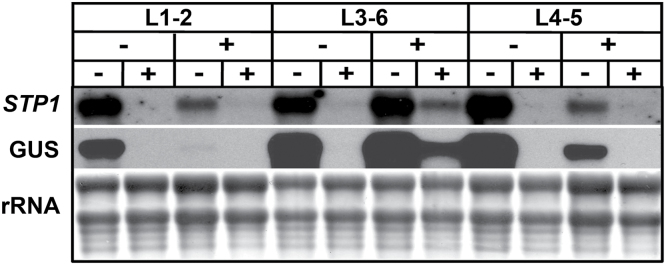
Sugar regulates *STP1* expression at a transcriptional level. Total RNA was isolated from 12-day-old plants from independent homozygous transgenic lines containing p*STP1*-2.4::GUS. Prior to the treatment, the plants were grown for 2 days in media depleted of sugar (–) or supplemented with 150mM Glc (+) and then transferred to media without (–) or with 150mM Glc (+) for 6h. Each lane contains 10 µg of total RNA, and the blot was hybridized with the *STP1* and GUS probes. The rRNA of the methylene blue-stained membrane is shown as a loading control. Membranes shown here are representative of three biologically independent experiments.

Our previous northern analysis demonstrated that the *STP1* transcript practically disappears after 1h of Glc addition (Fig. 1B), thus the activity of GUS was followed in the transgenic plants after the addition of 150mM Glc. In contrast to the RNA analyses noticeable differences in GUS accumulation in the Glc-treated plants were not observed prior to 12h of Glc exposure (data not shown). After 12h of Glc treatment a reduction in the GUS activity was observed in all the transgenic lines (Fig. 5B and E). This response is specific for Glc as it is not observed with isosmotic concentrations of Mtl (Fig. 5C and F), which display an undistinguishable GUS level compared to the one observed without the carbon source (Fig. 5A). The decrease in GUS expression in response to the presence of Glc was most prominent in the stomata and trichomes, where the defined patterns observed in these structures were basically lost (Fig. 5E and H). However, even after 12h of Glc treatment, considerable GUS activity was detected in the sugar-treated transgenic plants (Fig. 5) in contrast to the endogenous *STP1* RNA response. This apparent discrepancy might be explained because it has been shown that GUS activity persists for long periods beyond its actual promoter activity (Kavita and Burma, 2008). Thus, to unequivocally demonstrate whether the *STP1* promoter in these transgenic plants contains the elements responsible for regulation by sugar observed with the endogenous *STP1* gene, the expression of GUS and *STP1* endogenous transcripts in these lines was analysed by northern blotting. As shown in Fig. 6, high levels of the GUS transcript were detected in the transgenic plants that were transferred to media without sugar (–); this high expression was independent of the initial growing media prior to the transfer (with or without sugar). In contrast, the GUS transcript was barely detectable when these plants were transferred to media containing 150mM Glc (Fig. 6). This regulation was very similar to the one observed for the *STP1* endogenous transcript (Figs 1C and 6). From this analysis, we concluded that the *cis*-acting elements responsible for Glc regulation of the *STP1* gene are contained in the 2.4kb fragment, at least under the conditions analysed.

### Delimiting the *cis*-acting regions of the *STP1* promoter that respond to Glc.

**Fig. 7.**
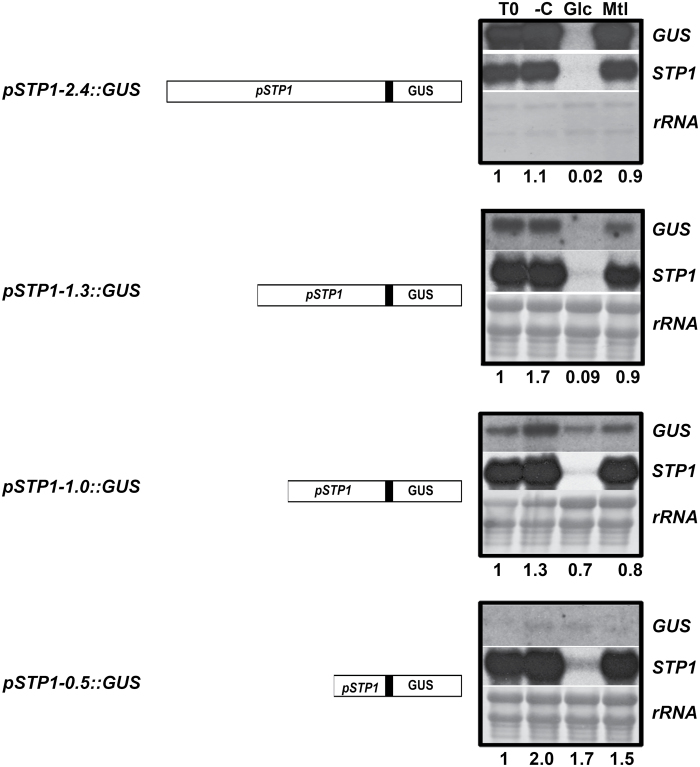
Deletion analysis of the *STP1* promoter region. Total RNA was obtained from representative 12-day-old transgenic homozygous lines containing 2.4, 1.3, 1, and 0.5kb of the upstream sequences of the *STP1* gene fused to GUS (p*STP1*::GUS), as indicated in each diagram. Plants were deprived of sugars for 2 days prior to being transferred to media without (–C), or with 150mM Glc or Mtl for 6h. T0 corresponds to the RNA from the plants prior to the transfer and was taken as the control. A total of 1 µg of total RNA was used for p*STP1*-2.4::GUS, whereas 20 µg was used for the other transgenic lines. Each blot was hybridized against the *STP1* and GUS probes as indicated in each deletion. The rRNA staining of the methylene blue membrane is shown as a loading control. The numbers at the bottom of each blot correspond to the level of the GUS transcript relative to the level found in the sample prior to the transfer (T0), which is taken as 1. Densitometric analyses were performed from at least two independent biological experiments.

In order to narrow down the specific elements involved in the sugar regulation of *STP1*, three consecutive deletions of the original 2.4kb upstream fragment were generated and fused to GUS; each containing 1.3kb, 1kb, and 0.5kb from the original fragment (Fig. 7). Independent transgenic lines were selected from each deletion based on their resistance to kanamycin, and the deletion size was corroborated by PCR (data not shown). Homozygous plants from a representative line were selected for each deletion, and the expression levels of *STP1* and *GUS* were analysed after sugar treatments. Treatments were conducted using 10-day-old plants starved of a carbon source for two days, after which the plants were transferred to different media (–C, Glc, or Mtl) for 6h. The expression level of the GUS transgene was compared to the level prior to the transfer (T0) in each deletion. We observed that the basal GUS transcript level (T0) was considerably lower in all deletions than the level observed in the 2.4kb fragment (Fig. 7). This result is particularly evident in the p*STP1-*1.3::GUS and p*STP1-*0.5::GUS constructs, indicating that important elements required for normal *STP1* expression level are localized between the deleted sequences in each case. However, independently of the basal transcript level (T0), the presence of 150mM Glc repressed the GUS transcript level in the p*STP1-*1.3::GUS deletion; the extent of repression was similar to the one observed with the initial p*STP1-*2.4::GUS construct (Fig. 7). In contrast, minor differences, if any, in the level of GUS transcript were observed in the two additional deletions (p*STP1-1::GUS,* and p*STP1-0.5::GUS*) in the presence of Glc (Fig. 7). Densitometric analysis from independent lines and independent experiments demonstrated that the presence of Glc results in a 98% reduction in the p*STP1-*1.3::GUS lines (*P* = 0.001) compared with the T0 control plants; only a 30% reduction was detected for the p*STP1*-1::GUS lines (*P* = 0.004) (Fig. 7). The efficiency of the treatments was corroborated by the response of the endogenous *STP1* transcript (Fig. 7). Together, these results demonstrate that Glc regulates *STP1* expression at the transcriptional level and that repression by sugar depends on *cis-*acting sequences contained within a 309bp fragment, which is localized between 1.3 and 1kb upstream of the *STP1* translational initiation codon (Fig. 7).

### *In silico* analysis of putative cis Glc-responsive elements in the 309bp *STP1* promoter fragment.

**Fig. 8.**
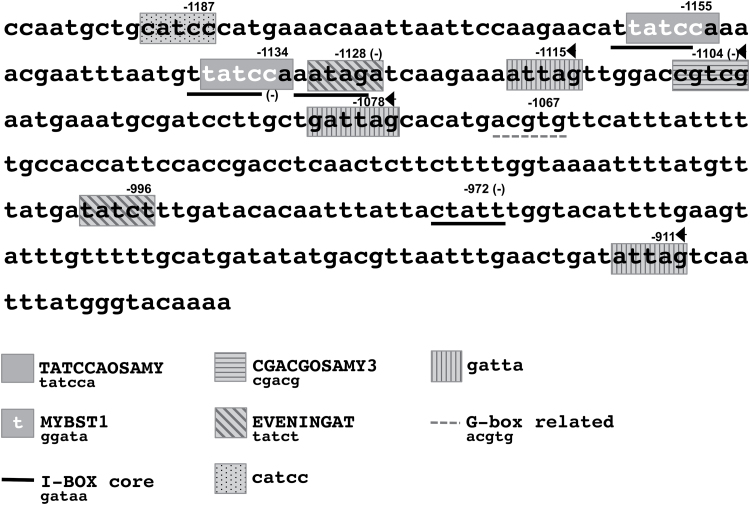
Putative sugar regulatory motifs in the 309bp region of the *STP1* promoter. The numbers indicate the position of the last base in each motif and refer to the translation initiation site of *STP1*. The overlapping elements are underlined. The arrowheads indicate elements found in reverse orientation and (–) in the complementary strand.

Previous studies have identified *cis-*regulatory elements for independent genes that participate in repression by sugars (Hwang *et al.*, 1998; Morita *et al.*, 1998; Toyofuku *et al.*, 1998; Tatematsu *et al.*, 2005). Thus, we performed an *in silico* analysis of the 309bp fragment and searched for motifs that are known to be involved in the repression by sugars. This analysis was performed using the PLACE database (http://www.dna.affrc.go.jp/PLACE/) and by also considering additional *cis* elements that were found to be overrepresented in the regulatory regions of sugar-repressed genes (Li *et al.*, 2006; Baena-Gonzalez *et al.*, 2007). This analysis revealed 17 potential elements in the 309bp fragment (Fig. 8 and Table 1) that belonged to eight different sugar motifs. One of the most interesting elements found in this region corresponds to the TATCCAOSAMY motif. This motif occurs twice in the 309bp *STP1* fragment (at –1134 and –1155 from ATG); the two instances are separated by 15bp (Fig. 7). The TATCCAOSAMY motif was originally found in the 5′ upstream regulatory region of the *α-Amy3D* gene from rice and has been demonstrated to be essential for the regulation of this gene by sugars (Lu *et al.*, 1998; Lu *et al.*, 2002). Part of the TATCCAOSAMY motif (TATCC) overlaps with two other elements (MYBST1 and I-BOX) in the complementary strand and in reverse orientation (Table 1). The I-BOX is found four times within this sequence (Fig. 8), but only two of these instances overlap with the TATCCAOSAMY motif. An additional element in this region was the CGACGOSAMY3 motif (Hwang *et al.*, 1998), which localized at –1104 in the *STP1* promoter (Fig. 8). This motif was also originally described in the promoter of the *α-Amy3D* gene and is required for the Glc repression of this gene (Hwang *et al.*, 1998). In addition, we identified seven sequences with homology to three elements that are overrepresented in the sugar-repressed genes in the microarray data reported by Li *et al.* (2006). Four of these sequences share homology with the GATTA motif, two with the EVENINGAT core element and one with the CATCC motif (Fig. 8 and Table 1).

**Table 1.** Known *cis*-acting elements involved in sugar repression in the 309bp fragment from the *STP1* promoter

Element	Sequence	Reference
CGACGOSAMY3	CGACG	Hwang *et al.*, 1998
TATCCAOSAMY	TATCCA	Lu *et al.*, 1998
SREATMSD	TTATCC	Tatematsu *et al.*, 2005
TATCCAYMOTIFOSRAMY3D	TATCCAY	Toyofuku *et al.*, 1998
MYBST1	GGATA	Baranowskij *et al.*, 1994
I-BOX core	GATAA	Manzara *et al.*, 1991
EVENINGAT core	ATATCT	Harmer et al., 2000; Li *et al.*, 2006
CATCC	CATCC	Li *et al.*, 2006
GATTA	GATTA	Li *et al.*, 2006
G-box related	ACGTG	Lu *et al.*, 1998; Baena-González *et al.*, 2007

Similar to *STP1*, the expression of the *DIN6* gene is strongly repressed by the presence of sugars and activated during sugar starvation (Baena-Gonzalez *et al.*, 2007). Thus, we decided to compare 309bp of the *STP1* fragment with the upstream sequence of *DIN6* gene. This analysis revealed only two motifs that were shared between these sequences: one of them is the TATCCAOSAMY motif, and the other is a sequence related to a G-box (ACGTG) (Fig. 8).

Finally, several members of the STP family are also repressed by sugars (Price *et al.*, 2004). Thus, we also searched for common motifs between *STP1* and three other known sugar-regulated members. For this analysis, the complete upstream intergenic regions of the *STP4* (At3g19930), *STP13* (At5g26340), and *STP14* (At1g77210) genes were compared against the 309bp fragment of the *STP1* promoter. Six out of the eight different motifs previously identified in *STP1* were also present at least once in the control regions of the other *STP* genes (Table 1). Interestingly, the CGACG and the TATCCAOSAMY motifs were found in *STP4* and *STP13* genes, but not in the *STP14* gene.

## Discussion

Sugars act as key regulators of gene expression by inducing or repressing the transcription of many genes (Koch, 1996). Many studies have contributed to understanding the mechanisms by which sugars regulate gene expression (Rolland and Sheen, 2005; Eveland and Jackson, 2012). Initial forward genetics studies were valuable for demonstrating the complexity of sugar signalling and provide evidence for the existence of multiple signalling pathways. However, genomics and system biology analyses have been crucial for demonstrating the effect of sugar availability on expression throughout the entire genome (Price *et al.*, 2004; Villadsen and Smith, 2004; Gutierrez *et al.*, 2007; Osuna *et al.*, 2007). The *STP* family is one of the gene families that has repeatedly been detected in genomic analyses as highly responsive to sugars (Price *et al.*, 2004; Villadsen and Smith, 2004). This family includes genes that encode low- and high-affinity monosaccharide transporters (Stadler *et al.*, 2003; Büttner, 2010; Slewinski, 2011). Compared to other members of the family, STP1 is a high-affinity H^+^/sugar cotransporter with the highest and broadest expression in *A. thaliana* (Büttner, 2010). Our data corroborated the findings that the expression of *STP1* is rapidly modulated by minor fluctuations in sugars levels (5mM Glc). This response contrasts with the response of other sugar-repressed genes, such as several photosynthetic genes, that require higher sugar levels and a longer time to affect the level of their transcripts (Acevedo-Hernandez *et al*., 2005). In addition to *STP1*, other members of the *STP* family have also been shown to be regulated by sugars; however, whether these involve common mechanisms is unknown.

Previous work demonstrated that *STP1* expression is induced by darkness and repressed by light in guard cells. This regulation has been suggested to be important for the import of carbon to these cells, particularly during dark periods (Stadler *et al.*, 2003). Our analysis with transgenic lines containing the p*STP1:*GUS fusion corroborated the view that one of the sites with major levels of GUS accumulation corresponds to the stomatal guard cells. Interestingly, this expression is notably decreased with exposure to Glc. Thus it is likely that at least part of regulation previously observed by light is linked to the sugar fluctuations in these cells during dark periods more than a direct downregulation by light. Since guard cells depend on sugar import to maintain their metabolism as they are unable to perform photosynthesis, it is likely that during dark periods the levels of phosphorylatable hexoses become very low and in consequence the expression of the *STP1* gene gets induced. Previous work has found an increase in *STP1* mRNA at the onset of the dark period. We could speculate that the decrease in sugar import as a result of the lack of photosynthetic activity, together with the start of starch breakdown that will supply carbon skeletons during the next hour, may mean that the actual intracellular phosphorylatable hexose levels are very low. The expression of a high-affinity sugar transporter such as *STP1* under these conditions is possibly important for transporting available external hexoses. Although additional experiments will be required to clarify these aspects, the sensitive and rapid response observed here for *STP1* expression is very well suited to ensuring proper sugar influx in response to minor fluctuations in sugar availability in guard cells as well as in other plant tissues. The other sites where high GUS expression was detected are the trichomes. However, the physiological reason for the requirement of this transporter in this type of specialized structure is less obvious and will require future analyses.

Considering the mechanisms, we believe that the rapid response of the *STP1* transcript to fluctuations in sugar levels suggests that some of the elements involved in the perception of the Glc signal should be present prior to the stimulus. This possibility agrees with the observation that the Glc repression of other *STP* genes *(STP14* and *STP4)* is normal in the presence of the translational inhibitor cycloheximide (Price *et al.*, 2004). Surprisingly, that study also found that the repression level of the *STP1* transcript appears to be less severe in the presence of cycloheximide. Thus, it is possible that the *de novo* synthesis of some of the *trans*-acting factors is required either to achieve full repression or to sustain this response (Price *et al.*, 2004). The present analysis also reveals that the half-life of the *STP1* transcript is apparently not very long; thus, the repression of the transcription level is reflected in the total transcript level within minutes of Glc addition.

Our analyses of *STP1* expression using different Glc analogues demonstrated that the signal that induces the repression of this gene is a phosphorylatable sugar. These data agree with previous reports that found that the non- or poorly phosphorylatable Glc analogues, such as 3-OmG and 6-deoxyglucose, did not change *STP1* expression (Cortes *et al*., 2003; Villadsen and Smith, 2004). Interestingly, our data also demonstrated that the sugar signal that modulates the repression of the *STP1* gene is independent of the HXK1 sensor. Therefore, a primary sensor different from HXK1 must perceive the phosphorylated sugars that initiate *STP1* regulation. In spite of the important efforts of many groups, still almost nothing is known about alternative receptors for sugar perception with the exception of the regulator of G protein (RGS1). RGS1 has been suggested to bind sugars and attenuate the cell division of the apical root meristem through its interaction with a heterotrimeric G protein independently of HXK1 (Chen *et al.*, 2003; Chen, 2008;). However, in this work, we demonstrated that RGS1 does not appear to play a major role in the sugar regulation of *STP1* because the repression of *STP1* by sugars is very similar to the repression in wild-type plants in the absence of this regulator.

The genome of most plants encodes various HXK-related genes in addition to HXK1: five in the case of *A. thaliana* and ten in rice (Granot *et al.*, 2013). Although some of these *HXK* genes have clear enzymatic activity (type A and B), others apparently lack such activity (*HKL*) and have been suggested to have regulatory functions (Xiao *et al.*, 2000; Rolland *et al.*, 2006; Karve *et al.*, 2008; Granot *et al.*, 2013). In fact, recent work provided evidence that different *HXK* genes have signalling roles in different plants. For example, several *HXK*-type B genes from potato and rice were able to complement the Glc sensitivity of the *gin2* mutant (Veramendi *et al.*, 2002; Cho *et al.*, 2009; Karve *et al.*, 2010). In addition, a signalling role was observed for some *HKL*-type genes in *A. thaliana* and *Physcomitrella* (Thelander *et al.*, 2005; Zhang *et al.*, 2010; Karve *et al.*, 2012). Whether any of the additional *HXK* genes (A, B, or HKL) have a role in the sugar regulation of *STP1* remains for future analysis.

Other players that have been shown to participate in sugar signalling are the SnRK1 and TOR kinases (Baena-Gonzalez and Sheen, 2008; Xiong *et al.*, 2013). SnRK1 kinase is highly conserved throughout the evolution of different organisms, including plants, and has been demonstrated to be crucial for energy homeostasis, such as carbon availability (Hardie *et al.*, 1998; Baena-Gonzalez, 2010). Importantly for the present study, alterations in *STP1* expression were reported in a microarray analysis from transiently overexpressing KIN10 protoplasts (Baena-Gonzalez *et al.*, 2007). *A. thaliana* contains two SnRK1 catalytic subunits (KIN10 and KIN11) that are partially redundant (Baena-Gonzalez *et al.*, 2007). However, it was not possible to analyse the double mutant due to its lethality; therefore, in this work, we explored the role of this kinase in the regulation of the *STP1* gene using the single *kin10* and *kin11* mutants (Polge and Thomas, 2007) as well as transgenic lines that overexpress KIN10. KIN10 has been reported to have the most notable activity of the two catalytic subunits (Jossier *et al.*, 2009). In this analysis, we did not observe major differences in the response of *STP1* to sugars in any of the various analysed mutants and lines. Thus, although the involvement of this kinase in the regulation of *STP1* cannot be completely ruled out, the only difference we observed is a slight reduction in the level of the *STP1* transcript in the overexpressing *KIN10* lines. Our data indicate that the participation of SnRK1, if any, in the regulation of *STP1* is minor.

None of the factors analysed so far play a major role in the regulation of *STP1*, suggesting the participation of novel factors in the regulation of this gene. Potential additional candidates include factors whose mutants display alterations in *STP1* expression. For example, in comparison to the wild-type plants, the *sweetie* mutant displays an upregulation of the *STP1* gene (Veyres *et al.*, 2008). *SWEETIE* encodes a novel protein of unknown function and is implicated in various processes, including sugar perception, senescence, ethylene biosynthesis, and abiotic stresses (Veyres *et al.*, 2008; Büttner, 2010). Misregulation of the *STP1* gene by sugars was also reported in *hsr* (*high sugar-response*) mutants. For several genes, these mutants displayed sugar hypersensitivity, and the elements that are affected in these mutants are good candidates for involvement in *STP1* sugar regulation. Unfortunately, the identities of the *HSR* genes are still unknown (Baier *et al.*, 2004).

In this work, we also demonstrated that sugar regulates the *STP1* gene at the transcriptional level, and this regulation is similar to that of the sugar-regulated genes *DIN6/ASN1* and *α-Amy3*, whose expression is also induced by sugar starvation and is repressed in its presence (Lam *et al.*, 1998; Lu *et al.*, 2002; Baena-Gonzalez *et al.*, 2007). Similarly to the *DIN6* and *DIN1* genes, the regulation by Glc of *STP1* is independent of the HXK1 pathway (Baena-Gonzalez *et al.*, 2007). These similarities support a common mechanism for the regulation of these genes by sugars. In the present analysis, we were able to delimit the *cis*-acting elements required for the *STP1* sugar repression within 309bp. Our *in silico* analyses showed the *cis*-acting elements that are common to the *STP1* 309bp sequence and the α-*Amy3* and *DIN6* promoters, including the TATCCA and the G boxes (Lu *et al.*, 2002; Baena-Gonzalez *et al.*, 2007). The TATCCA element (TATCCAOSAMY) was originally identified as the binding site of one MYB-type transcription factor (OsMYBS2) that is essential for the sugar regulation of the α-*Amy3* gene in rice (Lu *et al.*, 2002). Moreover, the arrangement of these elements in the *STP1* promoter (in tandem and separated by 15bp) is similar to that in the α-*Amy3* gene (Lu *et al.*, 1998). Thus, this sequence is an interesting candidate for involvement in the regulation of the *STP1* gene by sugars. MYB transcription factors are members of a large gene family in plants with more than a hundred members in *A. thaliana* (Dubos *et al.*, 2010). Two MYB genes in *A. thaliana* (At5g47390 and At5g61620) are the closest to the rice MYBS2 factor based just on protein sequence identity. However, the role of this putative orthologue requires further study.

The *STP14* gene does contain five TATCCA elements in the 5′ UTR that is shared with both *STP1* and *DIN6*. However, neither the TATCCAOSAMY nor the CGACGOSAMY3 motifs are present in the upstream sequence of the *STP14* gene; this gene and *STP1* are among the most sugar-repressed genes of the *STP* family (Price *et al.*, 2004). Thus, the contribution of any of these elements to the control by Glc of the *STP* genes must be determined in the future.

Finally, a low but reproducible increase in the *STP1* transcript at midday was reported and linked to a circadian regulation of this gene (Harmer *et al.*, 2000; Stadler *et al.*, 2003). This is an interesting aspect taking into account that one of the motifs present in the region responsible for sugar regulation includes the EVENINGAT element present in genes regulated by the circadian clock (Harmer and Kay, 2000).This element was also found overrepresented in the sugar-repressed genes in a microarray data reported by Li *et al.* (2006). It is possible that the expression of the *STP1* gene, like many other genes, might be subjected to multiple regulatory mechanisms, in addition to sugars. However, recent evidence supports the view that the levels of sugars directly influence the circadian regulation of many genes (Haydon *et al.*, 2013), supporting possible crosstalk between these regulatory mechanisms. Although that there is still not a direct probe to show that the EVENINGAT element might be directly involved in sugar regulation, this aspect is an interesting possibility that requires further exploration in the future.

## Funding

This work was supported by grants 154392 and 127546 from the Consejo Nacional de Ciencia y Tecnología (CONACyT) and IN208211-3 and IB200511 from DGAPA. DLAZ was supported by DGAPA with an undergraduate fellowship from grant IB200511.
